# The Common Task

**Published:** 1907-09-28

**Authors:** 


### Nursing Administration.

### THE COMMON TASK.

**Correspondence and Queries** for this section should be sent to the Editor of The Hospital, 28 Southampton Street, Strand, London, and marked "Nursing Administration."

### Medical Mission Work in India.

The motto of the Zenana Bible and Medical Mission Society is "The Women publish the tidings," and we note that this is well borne out in the recent report of work accomplished during the past year. Trained nurses are in great request for aiding in the medical missions, part of the endeavour being to train Indian women as nurses to labour among their own people. The society affords an advantageous medium through which those who desire to take up mission work in India may find their zeal turned to the best account.

### The Baby's Welcome.

A new movement in favour of promoting thrift among expecting mothers has been started in Wakefield, and seems likely to spread in many towns where an inadequate sense of the responsibilities of childbirth prevail. Charitable ladies are beginning to awake to the perception that more is needed to secure comfort for the newcomers than the bag of necessaries which too often acts as a direct incentive to the mother to omit any personal preparations. The district nurse penetrating into the homes at the critical period, and getting to all the secrets of its unthrift, has opened the eyes of the public to a hundred fresh theories. The mothers need teaching even more than linen, and the smallest article bought as the fruit of their own forethought is of more value than all the garments which are lent around the parish and get to be known as the badge of an indolent mother. The familiar mothers' meeting often does much to develop this sense of thrift, but it might undoubtedly do more. It might furnish patterns of garments suitable for babies, and thus relieve the mother of the onerous duty of preparing some fifteen or eighteen separate articles for the overburdened baby's use. It might have model cots on view, and arrange with dealers to supply them at small cost. It might furnish papers giving simple directions about feeding. It might invite the district nurse occasionally to come and give a demonstration on the art of washing a baby, or give a homely talk on some difficulties of feeding in connection with delicate babies. We notice that it is part of the Wakefield scheme to enlist the co-operation of district nurses, and we hope they will not stop short at collecting pennies from the mothers by laborious house-to-house visitation, but will take counsel with the superintendents as to the best methods of inspiring them with right principles of infant management.

### The Dinner-hour in Institutions.

It is curious, and not very creditable to the heads of institutions, that although it has now long been acknowledged that leisurely mastication is the first essential to digestion, meals are nowhere scrambled through with more indecent haste than in establishments dedicated to the cure of disease. The top speed necessary to satisfy the appetite in many hospitals is a standing menace to health, and the very complaints which the institution exists to cure may be observed in process of manufacture among the staff. Not only is the dinner-hour often reduced to twenty minutes by unpunctuality on the part of those who preside, or indifferent waiting, but bad organisation frequently prescribes that some of the nurses shall come short even of this bare minimum. The best that can be done under the circumstances is to drink the food, for eating which implies mastication demands time. Taking into consideration the moments spent in assembling, the intervals between courses, the delays of service, and the needs of late comers and early goers, it may be safely laid down that three-quarters of an hour is not too long to assign as the nominal dining hour if all are to have an opportunity of treating their digestive organs with respect. Punctuality and smart service may succeed in making a half-hour dinner tolerable, but any appearance of haste is entirely destructive of the right atmosphere which ought to prevail. We heard recently of a large institution where a scanty quarter of an hour was all that was available for the principal meal of the day, and we wish that we could believe that this were an isolated instance of a very bad custom.

